# ‘My life is a mess but I cope’: An analysis of the language children and young people use to describe their own life-limiting or life-threatening condition

**DOI:** 10.1177/02692163241233977

**Published:** 2024-03-04

**Authors:** Katherine Bristowe, Debbie Braybrook, Hannah M Scott, Lucy Coombes, Daney Harðardóttir, Anna Roach, Clare Ellis-Smith, Myra Bluebond-Langner, Lorna Fraser, Julia Downing, Fliss Murtagh, Richard Harding

**Affiliations:** 1 Cicely Saunders Institute of Palliative Care, Policy & Rehabilitation, King’s College London, London, UK; 2 Royal Marsden NHS Foundation Trust, Sutton, UK; 3 Great Ormond Street Institute of Child Health, Faculty of Population Health Sciences, London, UK; 4 Louis Dundas Centre for Children’s Palliative Care, University College London, London, UK; 5 Rutgers University, New Brunswick, NJ, USA; 6 International Children’s Palliative Care Network, Kampala, Uganda; 7 Wolfson Palliative Care Research Centre, Hull York Medical School, Hull, UK

**Keywords:** Communication, paediatrics, palliative care, linguistics, qualitative research

## Abstract

**Background:**

Children and young people with life-limiting and life-threatening conditions have multidimensional needs and heterogenous cognitive and communicative abilities. There is limited evidence to support clinicians to tailor their communication to each individual child.

**Aim:**

To explore the language children and young people use to describe their own condition, to inform strategies for discussing needs and priorities.

**Design:**

Positioned within a *s*ocial constructivist paradigm, a secondary discourse analysis of *s*emi-structured interview data was conducted incorporating the discourse dynamics approach for figurative language.

**Setting/participants:**

A total of 26 children and young people aged 5–17 years with life-limiting or life-threatening conditions (6 cancer; 20 non-cancer) were recruited from nine clinical services (six hospitals and three hospices) across two UK nations.

**Results:**

The language children and young people use positions them as ‘experts in their condition’. They combine medical terminology with their preferred terms for their body to describe symptoms and treatments, and use comparatives and superlatives to communicate their health status. Their language depicts their condition as a ‘series of (functional and social) losses’, which single them out from their peers as ‘the sick one’. Older children and young people also incorporate figurative language to expand their descriptions.

**Conclusion/discussion:**

Children and young people can provide rich descriptions of their condition. Paying attention to their lexical choices, and converging one’s language towards theirs, may enable more child-centred discussions. Expanding discussions about ‘what matters most’ with consideration of the losses and differences they have experienced may facilitate a fuller assessment of their concerns, preferences and priorities.

**What is already known about the topic?**

- Children and young people living with life-threatening and life-limiting conditions have multidimensional needs and heterogenous cognitive and communicative abilities.
- A child-centred approach is required to ensure the child or young person’s best interests are paramount, and that their views and priorities are taken into account.
- Clinicians report challenges in shaping their communication to the needs of each child or young person.

**What this paper adds?**

- The language children and young people use positions them as experts in their condition, providing detailed descriptions of symptoms and holistic assessments of their health status.
- Older participants also use figurative language to further explain their symptoms and emotional responses.
- The lexical choices made by children and young people frame their condition as a series of losses, which contribute to them being perceived as ‘the sick one’.

**Implications for practice, theory and policy**

- Children and young people can provide rich descriptions of their condition and its impact upon their life.
- Paying attention to these lexical choices, and converging one’s language towards theirs, may enable child-centred discussions about preferences and priorities.
- Expanding discussions about ‘what matters most to you’ with consideration of the losses and differences children and young people experience may enable a fuller assessment of preferences and priorities.

## Introduction

An estimated 21 million children and young people globally are living with a life-limiting or life-threatening condition that may shorten their life.^1^ They represent a chronologically and developmentally heterogenous group ranging from infancy, through childhood and into adolescence. Many children and young people living with life-limiting or life-threatening conditions are not able to communicate verbally. The highest prevalence of these conditions is found in children under 1 year (226.5/10,000), and the most prevalent causes are congenital (27.2/10,000) and neurological (10.8/10,000) conditions, which are often associated with severe disability, cognitive impairment or developmental delay.^2^ Many studies therefore draw on the views and experiences of proxies, family members and healthcare professionals,^3^ rather than from children and young people directly.

Some children and young people however can report on their own problems and concerns, and contribute to research, as evidenced by the wealth of literature on the experiences of children with chronic conditions, including some cancers. They describe a desire to live an everyday life, but struggle with isolation and loneliness.^4^ They also feel powerless and invisible when conversations are had about them, rather than with them.^5^ Children facing a potentially shortened life also value the opportunity to engage in age appropriate activities and pursuing normality, but alongside existential concerns related to the experiences and milestones they may never achieve.^6–8^ As such clinicians working with children and young people require specific evidence to support best practice in the context of a potentially shortened life.

Clinicians have a legal and ethical imperative to enable patients to be participants in their healthcare.^9^ Person-centred care is holistic, multidimensional and respects the person’s values, priorities and perspectives, by actively involving them in their treatment decisions.^10^ Child-centred care draws on the same tenets but recognises that the child exists within a dynamic familial and social context, but has their own views, priorities and preferences which must be recognised and respected, and which may differ from those of their parents.^11^ The child and their family are seen as partners in the care, and the best interests of the child remain paramount.

Although clinicians are skilled at delivering holistic care, they report challenges shaping their communication to the needs of children and young people with life-limiting or life-threatening conditions,^12^ as many such conditions interrupt a child’s development making assessments about age-appropriate language more difficult. In these instances clinicians may defer to the family members rather than the child,^13^ resulting in a reduction of child-centredness. It is recognised that children and young people are more likely to engage in care discussions if directly invited to do so.^14^ In addition, sociolinguistic theories posit that accommodative behaviours, that enable convergence of communication features, may aid social interaction.^15^ As such, paying attention to how children and young people describe their own condition and the linguistic features *they* use, may support clinicians in adjusting their language appropriately and delivering child-centred care.

This work forms part of a programme of research to develop and validate a person-centred outcome measure (C-POS) for children and young people with life-limiting or life-threatening conditions.^3,6–8,16,17^ As well as providing vital person-centred outcome data, tools such as these support clinicians in their interactions by prompting discussion of common problems and concerns. This study aimed to explore how children and young people describe their own condition and the linguistic features *they* use, to inform strategies for communicating with them about their needs and priorities.

## Methods

### Study design

This qualitative study is positioned within a social constructivist paradigm^18^ which recognises that knowledge and human experience are socially situated and constructed in interaction with others. As this study sought to explore the language children and young people use to describe their own illness experience, to inform strategies for engaging them about their priorities, a paradigm that recognises the interactive nature of healthcare delivery was most appropriate. This study represents a secondary analysis^19^ of data collected to inform the development of a paediatric palliative care outcome measure.^7^ This supplementary secondary analysis falls within the aims and objectives of the original study, but provides a more in-depth analysis of one emergent feature of the data only partially reported in the primary study, namely the language children and young people use themselves to describe their illness experience. This study is reported in accordance with the Consolidated Criteria for Reporting Qualitative Studies (COREQ).^20^

### Setting

Children and young people were recruited from nine clinical services (six hospitals and three hospices) across two UK nations.

### Inclusion criteria

Children and young people aged 5–17 years diagnosed with at least one life-limiting or life-threatening condition.

### Exclusion criteria

Children and young people unable to communicate sufficiently to participate in a qualitative interview aided by play and drawing and with support from their parents as required; who speak a language not supported by NHS translation services; currently enrolled in another research study; or unable to consent or assent to participate.

### Sampling

Children and young people were purposively recruited to enable a breadth of characteristics by age and condition. Recruitment continued until pragmatic saturation, or information power, was achieved and sufficient information had been attained to meet the study aims.^21^

### Recruitment

Potentially eligible children and young people were identified weekly at multidisciplinary team meetings, ward rounds and outpatient appointments. Children and young people and those with parental responsibility were approached by the clinical team to introduce the study. If interested, they were provided with an information sheet outlining the purpose of the study and given time to decide whether they were interested in participation. Four sets of study materials were developed for children and young people (ages 5–7 years, 8–10, 11–15 and 16–17). Chronological age does not always equate with developmental age for children and young people with life-limiting and life-threatening conditions, so parents or caregivers were asked to select the most appropriate one for their child. Those who agreed were put in contact with the research team for further information, and if they remained interested an interview was arranged. Participants aged 16 years or older provided written informed consent. Participants under 16 provided assent, and consent was provided by those with parental responsibility.

### Data Collection

Semi-structured interviews were conducted by three female researchers: DB (experienced qualitative researcher), LC (palliative care nurse, new to qualitative research) and AR (new to qualitative research), with support provided by KB (qualitative methodologist). Interviews were conducted face-to-face in the participant’s preferred location. Following the introduction of COVID-19 social distancing restrictions, potential participants were offered the opportunity to participate via telephone or video call. Four interview topic guides were also developed for the different age groups (see Supplemental Materials). The structure for these was informed by the WHO definition of paediatric palliative care^22^ and a systematic review of symptoms and concerns of children and young people with life-limiting or life-threatening conditions.^3^ Where children and young people were accompanied by a parent or caregiver during the interview they were asked to assist in supporting their child to participate. Interviews commenced with the researcher introducing themselves and their role in the study, followed by basic demographic questions, exploration of interests and hobbies, followed by a question asked about the impact of the condition on their life, and whether they had opportunities to talk about what matters to them. The interviewers were led by the participants, allowing them to talk about what matters to them, whilst probing key areas informed by the WHO definition of paediatric palliative care. Interviews were audio-recorded, transcribed verbatim and pseudonymised. A reflexive diary was kept to capture emergent themes and reflections on the interview itself. It was not possible to return transcripts or summarised findings to participants for checking. Throughout the data collection period the team (DB, LC, AR and KB) met frequently to review transcripts, discuss interview findings and make any necessary adjustments or refinements to the topic guide.

### Ethical considerations

There is a growing body of evidence that demonstrates children and young people’s willingness to participate in research in the face of advanced illness, viewing it as a positive and rewarding experience.^1^ However, as potentially vulnerable people, the following steps were taken to minimise the risk of participation. Following advice from their parent/caregiver, potential participants had the study explained to them at an appropriate time and using language aligned to their communicative abilities. They were given a minimum of 24 h to decide whether to participate. To minimise potential burden and distress, researchers gave information about the interview content prior to the consent process, and were cautious to ensure no clinical information was disclosed that could cause additional distress. Researchers were trained to identify signs of distress, and to acknowledge this, and gave opportunities to pause or terminate the interview as needed. All interviews concluded with a 10 min debrief after the interview had finished to assess the impact of the interview upon participants. Any apparent distress was responded to, and participants were referred to the clinical team or community support sources as required.

### Analysis

An inductive discourse analysis^23^ was conducted in two stages to explore the language children and young people use to describe their condition and to identify common patterns, features and themes within the lexical choices. The first stage of the discourse analysis sought to extract and develop a taxonomy of the words and phrases used by the participants to describe the impact of the condition upon them, specifically their symptoms and concerns. These were categorised within the domains of palliative care (physical, psychological, social and spiritual), normalcy and life status assessments. The second stage sought to identify figurative, including idiomatic, descriptions of their illness experience within these same domains. Identification of figurative language drew on the Discourse Dynamics Approach^24^ which focusses on identifying ‘vehicle terms’ in the data, where the meaning in context stands out due to its figurative usage in comparison to the more familiar basic (contemporary) meaning.^25^

## Results

### Participants

Twenty-six children and young people (see Table 1 for participant and interview characteristics) participated in a single semi-structured interview (April 2019–September 2020).

**Table 1.** Participant characteristics.

Age	Mean 12 years (range 5–17)
Aged 5–7 years	*n* = 3
Aged 8–10 years	*n* = 7
Aged 11–15 years	*n* = 12
Aged 16–17 years	*n* = 4
Gender	
Male	*n* = 9
Female	*n* = 17
Condition (only ICD-10 chapter headings are reported to preserve anonymity)	
Gastrointestinal	*n* = 10
Cancer	*n* = 6
Neurological	*n* = 5
Congenital	*n* = 3
Metabolic	*n* = 1
Respiratory	*n* = 1
Present during interview	
Participant and researcher only	*n* = 3
Participant, researcher and parent	*n* = 18
Participant, researcher and sibling	*n* = 1
Participant, researcher, sibling and parent	*n* = 1
Participant, research and paid caregiver	*n* = 3
Interview duration	Mean 37 min (range 12–81 min)
Location of interview	
Hospital or hospice	*n* = 16
Home	*n* = 6
Video call	*n* = 3
Phone call	*n* = 1

### Findings

The lexical choices made by the children and young people have been categorised into three themes. They used language which: positioned themselves as ‘an expert in their own condition’, presented the ‘condition as a series of losses’ and communicated the challenges of ‘being the sick one’. Each of the themes are presented below with examples from the data. NB. An additional table (Table 2) has been provided which presents a comparison of the lexical choices made by younger (aged 5–10 years) and older (aged 11–17 years) participants (see Supplemental Materials). *Being an expert in your own condition*

Participants demonstrated considerable insight into and knowledge of their condition, and provided rich descriptions of the impact it had upon them. They were able to describe symptoms or treatments in detail identifying parts of the body, and demonstrating their preferred terms in so doing (e.g. *belly’, ‘stomach’, ‘throat’, ‘bottom’, ‘butt’, ‘liver’, ‘spleen’*).

‘Participant: *That’s a scar because they cut my belly open and erm. . .they. . .and I normally call it a shark bite now and I. . . . . .and I pretend to. . .it. . .being eaten. . .*

Interviewer: *(laughter) well that’s pretty. . .that’s quite a cool story isn’t it?*

Parent: *It is isn’t it (laughter)*

Interviewer: *Do you know why they cut your belly open?*

Participant: *Because I had a liver transplant’*

(Participant 11, aged 5, gastrointestinal condition)

The older participants in particular also used adjectives to describe the nature of symptoms particularly pain (e.g. ‘achy’, ‘burning’ and ‘sharp’). Across all age groups they utilised complex medical terminology (e.g. ‘cannula’, ‘septic’, ‘biopsy’, ‘bronchoscopy’, ‘diuretic’ and ‘portacath’), which they had incorporated into their lexicon to describe symptoms, tests, diagnoses and treatments.

Participant: *Umm. . .I don’t like operations*

Interviewer: *Okay, have you had to have any operations?*

Participant: *Yes*

Interviewer: *Which operations have you had to have?*

Participant: *Umm. . .well I’m. . .I’ve had my portacath changed. I’ve had bronchoscopies. . .’*

(Participant 14, aged 12, respiratory condition)

They demonstrated the ability to create comparatives and superlatives to describe symptom severity (e.g. ‘a bit’, ‘a little’, ‘a lot’, ‘worst’, ‘weaker’ and ‘get worn out quicker’). However, they were also able to provide a holistic assessment of their health status, and compare this to previous timepoints (e.g. ‘poorly’, ‘really poorly’, ‘feeling well’, ‘not well enough’, ‘better’ and ‘worse’).

*‘Well erm, on the week where I have chemo, usually I feel a bit, bit erm rubbish really. Because it’s sort of, even though sometimes I might not be actually sick, it just it leaves that feeling you know, so sometimes I just might not be 100%. So it sort of it means I can’t, you know, go out as much. I try and get out on walks and things like that when I can, when I’m feeling up to it, but it means that I can’t really meet up with friends, or you know, I might not feel up to doing anything like that. So it sort of reduces the chance of me doing anything like that really’*

(Participant 24, aged 14, cancer)

Older participants also demonstrated their familiarity with figurative expressions, predominantly similes rather than metaphors and used them to expand on particular aspects of their experience. This included descriptions of pain being ‘like a knife going through’ and feeling ‘like a hawk’, overseeing their care, making sure treatments were being administered correctly.

*‘I am like a hawk when I’m in there. I am always like watching. I am like, “are you doing it right, are you doing it right? Are you actually flushing it with the right thing? Is it like that?” uhhhh ((sighing)). . .’*

(Participant 8, aged 14, congenital condition).

Another participant combined figurative expressions of a ‘tight band’ squeezing them with medical terminology to describe the impact of their ‘portal hypertension’. These figurative expressions give insights into the participants’ awareness, and the necessary vigilance related to their condition and treatments.

*‘Umm. . .it’s like up here [pointing to under ribs]. Like it’s like a tight band squeezing me. . .yeah, so its like right upper quadrant across to the left. So yeah and then because obviously my spleen’s enlarged as well because I have. . . portal hypertension. So, it’s like my spleen’s enlarged and so is my liver.’*

(Participant 17, aged 13, gastrointestinal condition)

Many older participants displayed a degree of acceptance or ambivalence when talking about their condition, feeling that it had become or always had been, the norm for them. They also demonstrated their acceptance of their condition through idiomatic phrases (e.g. ‘I can live with that’, ‘my life is a mess, but I cope’), and figurative expressions including examples related to toys and video games.

*‘All human beings have some purpose in their life, at a specific age, and no one can predict the future, I mean maybe they can when they’re 20 years later but you never know because it’s your life and you can’t see outside. You can only look the direction forwards, right and left because you can’t look backwards and zoom out on yourself like a videogame or anything.’*

(Participant 1, aged 13, cancer)

#### The condition as a series of losses

Despite a degree of acceptance, many participants were severely affected by the impact of their condition. When asked about the impact this was frequently described in terms of the deficits incurred, and the things that they cannot do or miss out on. For the younger participants this tended to take the form of specific description or physical impacts of their condition and how it affected their mobility and function (e.g. ‘couldn’t move my arm’, ‘can’t use my arms and legs’ and ‘trouble keeping up with my schoolwork’), and the friends and family that they missed seeing (‘I miss Daddy and [dog]’, ‘I miss all my friends’). Whereas for the older participants the broader impact on relationships and experiences was emphasised (e.g. ‘can’t do as much as other people’, ‘I miss out’).

*‘I’d just say, erm because my friend she’s been texting me and I, I just feel like I miss out like on stuff at school, like friendship-wise. Like you know, ‘cause like they’ll always be like, oh a new story and I’ll be like, ‘Oh what are you guys talking about?’ and they’re like, and they’re like, ‘Oh yeah when you weren’t here and stuff like this happened’ and like, they get to do like, sometimes they get to do like [pause] like fun stuff and like I get to miss out on it. . . Yeah. I just think, I just miss like [pause] the environment of school and like, talking with people, because it gets lonely as well.’*

(Participant 12, aged 15, gastrointestinal condition)

For the oldest participants, the loss of expected independence was particularly challenging (e.g. ‘can’t go to school by myself’), as was the loss of any form of privacy due to the severity of their condition.

*‘Well, erm. . . its, it doesn’t really give me, I can’t really have that much privacy because we don’t know whether or not I’m going to have a seizure or not and when I do have a seizures it’s a full body, moving side to side and I could bang my head or like, there’s another seizure I have which is like a drop attack, its just like I’ve just fainted and I just fall back.’*

(Participant 25, aged 17, cancer)

These impacts that affected the participant’s ability to engage with markers of maturity, or in ‘normal’ activities were particularly hard for them. One prominent example of this was in relation to food and eating. Participants described ‘not having an appetite’, ‘not wanting to eat’ and not being ‘allowed to eat certain things’.

Interviewer: *Yeah. And when you are feeling ill does that affect your eating at all?*

Participant: *I don’t want to eat.*

Interviewer: *You don’t want to eat. Ok.*

Participant: *I think sometimes just seeing food makes me feel horrible.*

(Participant 3, aged 12, cancer)

For the older participants losses or deficits were also frequently described in the context of hobbies and activities that they were previously able to enjoy.

*‘I’ve been wearing my splint mostly everyday and it. . .it helps a bit but not as much as I want it to help. I just want to be able to take the further step to get back to like me playing football.’*

(Participant 23, aged 17, cancer)

This highlighted the differences between them and their peers or their former selves, resulting in a loss of confidence and inability to fit in with their peers.

*‘I mean confidence wise it makes me feel bigger because I’m. . .I’m more enlarged around the middle and it’s just like my self-confidence goes. . . it’s quite low because obviously I can’t do. . .like wear lots of other things that people can wear. . . Well I can but I just don’t feel as confident in them’*

(Participant 17, aged 13, gastrointestinal condition)

Although less commonly expressed, some younger participants also described the emotional impact of these markers of difference.

‘*‘Like I could choke on baked beans because of my swallowing tube. And my feelings are happy when people are nice and caring and sad when people are mean and tease me’*

(Participant 13, aged 10, gastrointestinal condition)

*Being ‘the sick one’*

The sense of being different because of their condition resulted in feelings of exclusion and othering for our participants. The descriptions of ‘being the sick one’ or ‘the one that has something’ was often constructed through comparison with others or their past self, and how they wanted to be perceived.

*‘I mean. . . I was quite separate, so like during my treatment I didn’t want anybody to come and see me because I was, my thought was “I don’t want them to see me as the sick (Participant name) and the one that isn’t able to do stuff”. . . I want them to remember me as the girl that they used to hang out with and stuff. So that’s, I was pretty firm on them not really coming to the hospital. . .’*

(Participant 25, age 17, cancer)

The impact of the condition on their appearance, and how they were perceived by others, was particularly challenging for participants of all ages. When asked about their symptoms and concerns, visible symptoms were frequently described, in terms of markers of difference or illness (e.g. ‘eyes went yellow’, ‘lost my hair’, ‘in a wheelchair’, ‘lost the lower part of my leg’ and ‘my tummy was big’). In addition, the impact of their condition on growth and stature compared to others was also challenging.

‘Interviewer: *Is he your big brother?*

Participant: *Yes. No, I am the big brother. . . But he’s taller*

Parent: *. . .he was small because . . . when he was first born he was on hospital for the first year. . . so, he had his transplant four years ago and like only started eating and drinking since then.’*

(Participant 15, aged 9, gastrointestinal condition)

Being ‘the sick one’ was also described in relation to the enduring presence of medical interventions in their lives including frequent contact with hospitals and healthcare providers (e.g. ‘have operations and stuff’, ‘have to go to hospital’ and ‘see the physios’). Of particular prominence were invasive or painful procedures (e.g. ‘finger pricker thing’, ‘swallowing tube’, ‘injections’ and ‘horrible medicines’), which were associated with fear, discomfort and pain and heightened emotions.

‘Participant: *Sometimes I have to get IVs. . .*

Interviewer: *Yeah and do you like it when you have an IV?. . .*

Participant: *Nooo, they get sore . . .*

Interviewer: *And then, how about how you feel inside? So do you ever feel worried about anything [participant name]?*

Participant: *Kind of. . .*

Interviewer: *No, do you ever feel nervous?*

Participant: *((pause)) no. but I do feel nervous when I get my IVs in*

Interviewer: *When you get your IV in yeah*

Participant: *I always start to cry*

Interviewer: *Yeah and what happens when you start crying?*

Participant: *Normally my mummy says stop crying, it’s okay, it’s just a needle*

Interviewer: *Yeah. And does that make you feel any better?*

Participant: *No’*

(Participant 22, aged 8, congenital condition)

Journey metaphors were commonly used by older participants in relation to their illness experience (e.g. ‘made me go down hill’, ‘you’re in this boat, you’re in this ocean of emotions’, ‘get back into my stride’ and ‘take a step back’), particularly in descriptions of exacerbations and recurrences, and to explain their emotional response to their condition. Participants also used idiomatic expressions to push back against their position as ‘the sick one’.

*‘But, for me, my body doesn’t feel the same as is used to but it’s still you or me or yourself, you’re not in a different person’s body. You’re in your own body, you haven’t gone anywhere, you’re still in there but with a different feeling.’*

(Participant 1, Aged 13, cancer)

## Discussion

### Main findings

Children and young people make lexical choices that contribute to patterns in their language use. Firstly, they present as experts in their condition, providing detailed descriptions of symptoms, using adjectives and medical terminology, alongside their preferred terms for their body. They provide assessments of their health status utilising comparatives and superlatives, and older children and young people expand their descriptions using figurative language. Secondly, they describe their condition as a series losses of functional, social and normal activities and for older children and young people, losses of independence and privacy. Thirdly, this focus on what they cannot do contributes to descriptions of themselves as ‘the sick one’, when compared to others or their past selves, resulting in distress and loss of confidence.

### What this study adds?

Clinicians have a legal and ethical imperative to enable children and young people to be participants in their healthcare,^9^ and effective communication is central to this. A qualitative systematic review of effective healthcare communication with children and young people reported four considerations when tailoring communication to the individual children and young people: giving appropriate and timely medical information, focussing on the person, utilising the most appropriate method of (e.g. creative, talking and written) and style of communication (promoting involvement and honesty), and engaging with the parents.^26^ However, such broad recommendations fall short of providing clinicians with the fundamentals of how to construct the interaction.

Previous research has evidenced that, when explored using developmentally appropriate methods, children and young people can describe their own symptoms and self-management strategies.^27^ The present study extends this knowledge through in-depth analysis of the lexical features they use. Children and young people are capable of adopting the language of their environment, in this case medical terminology, and juxtaposing it within their own lexicon, to provide rich descriptions. As such, clinicians should ensure questions and discussions are directed towards the child in the first instance, and that they are encouraged to respond themselves, to avoid loss of agency and feelings of invisibility and powerlessness.^5^ In addition, children and young people value different communication features and strategies to their parents.^28^ Therefore, being mindful of the language choices children and young people make, checking in on their own understanding and usage of terms, and accommodating language choices by mirroring these features in one’s own lexicon, may strengthen clinical relationships.^17^

It has been recognised that goals of care, quality of life, living life to the fullest and comfort may be useful concepts to structure paediatric consultations around.^29^ However, these are quite abstract, and may not be the most accessible constructs. The children and young people in this study frequently talked about their condition as a series of losses, and were particularly focussed on the differences between them and their peers. Balancing questions about ‘what matters most to you’ with sensitive exploration of losses and differences may elucidate a more complete picture of experience, preferences and priorities. Recent research has enabled a more theoretically informed understanding of person-centred care for adults facing serious illness.^30^ This study makes important first steps in constructing a theoretical understanding of child centred palliative care, and offers insights and communication strategies to achieve this: recognising, exploring and mirroring their lexical choices and sensitively enquiring about losses and differences alongside preferences and priorities to enable a fuller assessment.

### Strengths and limitations of the study

This study contributes to the small but growing literature that draws directly on the views and experiences of children and young people living with life-limiting or life-threatening conditions. A strength of this study is the diversity of the sample in terms of age and condition, as much of the previous research has either relied on proxy reporting, retrospective reporting by adults who experienced illness in childhood, or has focussed primarily in cancer.^6^ This study supports the growing body of evidence that demonstrates that many children and young people can, and want to, participate meaningfully in research. However, the findings are not necessarily transferable to the views and experiences of children and young people with developmental delay, cognitive impairment or who are non-verbal. A limitation of this study is that a large proportion of the sample were children and young people living with gastrointestinal conditions, who represent a minority of the overall population of children and young people living with life-limiting or life-threatening conditions. A second limitation of the study is that we did not collect data on ethnicity of participants. Future research should include ethnicity in purposive sampling criteria, including children and young people communicating in an additional language or through an interpreter. Specifically, further research exploring the needs, experiences and communication preferences of black, Asian and Bangladeshi children and young people, who represent a disproportionate number of children and young people affected by life-limiting or life-threatening conditions,^5^ is vital.

## Conclusions

Children and young people are experts in their condition, and demonstrate this through the lexical choices they make when describing their experiences. They can express holistic needs, give specific descriptions of symptoms, form comparatives and superlatives and describe changes in health status. Older children and young people also use figurative language to enhance their communication. To facilitate child centred discussions about preferences and priorities, we recommend clinicians should sensitively frame conversations about losses and differences, as well as what matters most to children and young people, to elicit detailed descriptions of symptoms and concerns. Alongside this, careful observation of, and convergence towards, children and young people’s language choices may enhance child centred care discussions.

## Supplemental Material

sj-pdf-1-pmj-10.1177_02692163241233977 – Supplemental material for ‘My life is a mess but I cope’: An analysis of the language children and young people use to describe their own life-limiting or life-threatening condition Supplemental material, sj-pdf-1-pmj-10.1177_02692163241233977 for ‘My life is a mess but I cope’: An analysis of the language children and young people use to describe their own life-limiting or life-threatening condition by Katherine Bristowe, Debbie Braybrook, Hannah M Scott, Lucy Coombes, Daney Harðardóttir, Anna Roach, Clare Ellis-Smith, Myra Bluebond-Langner, Lorna Fraser, Julia Downing, Fliss Murtagh and Richard Harding in Palliative Medicine

sj-pdf-2-pmj-10.1177_02692163241233977 – Supplemental material for ‘My life is a mess but I cope’: An analysis of the language children and young people use to describe their own life-limiting or life-threatening condition Supplemental material, sj-pdf-2-pmj-10.1177_02692163241233977 for ‘My life is a mess but I cope’: An analysis of the language children and young people use to describe their own life-limiting or life-threatening condition by Katherine Bristowe, Debbie Braybrook, Hannah M Scott, Lucy Coombes, Daney Harðardóttir, Anna Roach, Clare Ellis-Smith, Myra Bluebond-Langner, Lorna Fraser, Julia Downing, Fliss Murtagh and Richard Harding in Palliative Medicine

sj-pdf-3-pmj-10.1177_02692163241233977 – Supplemental material for ‘My life is a mess but I cope’: An analysis of the language children and young people use to describe their own life-limiting or life-threatening condition Supplemental material, sj-pdf-3-pmj-10.1177_02692163241233977 for ‘My life is a mess but I cope’: An analysis of the language children and young people use to describe their own life-limiting or life-threatening condition by Katherine Bristowe, Debbie Braybrook, Hannah M Scott, Lucy Coombes, Daney Harðardóttir, Anna Roach, Clare Ellis-Smith, Myra Bluebond-Langner, Lorna Fraser, Julia Downing, Fliss Murtagh and Richard Harding in Palliative Medicine

sj-pdf-4-pmj-10.1177_02692163241233977 – Supplemental material for ‘My life is a mess but I cope’: An analysis of the language children and young people use to describe their own life-limiting or life-threatening condition Supplemental material, sj-pdf-4-pmj-10.1177_02692163241233977 for ‘My life is a mess but I cope’: An analysis of the language children and young people use to describe their own life-limiting or life-threatening condition by Katherine Bristowe, Debbie Braybrook, Hannah M Scott, Lucy Coombes, Daney Harðardóttir, Anna Roach, Clare Ellis-Smith, Myra Bluebond-Langner, Lorna Fraser, Julia Downing, Fliss Murtagh and Richard Harding in Palliative Medicine

sj-pdf-5-pmj-10.1177_02692163241233977 – Supplemental material for ‘My life is a mess but I cope’: An analysis of the language children and young people use to describe their own life-limiting or life-threatening condition Supplemental material, sj-pdf-5-pmj-10.1177_02692163241233977 for ‘My life is a mess but I cope’: An analysis of the language children and young people use to describe their own life-limiting or life-threatening condition by Katherine Bristowe, Debbie Braybrook, Hannah M Scott, Lucy Coombes, Daney Harðardóttir, Anna Roach, Clare Ellis-Smith, Myra Bluebond-Langner, Lorna Fraser, Julia Downing, Fliss Murtagh and Richard Harding in Palliative Medicine
