# Supplementary material for: ‘My life is a mess but I cope’: An analysis of the language children and young people use to describe their own life-limiting or life-threatening condition
Source: Palliat Med. 2024 Mar 4;38(3):379–88. doi: 10.1177/02692163241233977 (PMC10973786; doi:10.1177/02692163241233977)
Supplement: sj-pdf-1-pmj-10.1177_02692163241233977 – Supplemental material for ‘My life is a mess but I cope’: An analysis of the language children and young people use to describe their own life-limiting or life-threatening condition [file sj-pdf-1-pmj-10.1177_02692163241233977.pdf]

Table 2: Results of analysis presented by age

Theme	Younger Participants (5-10 years)	Older Participants (11-17 years)
Being an expert in your own condition		
Parts of the body	*'Belly', 'tummy', 'stomach', 'throat', 'bottom', 'butt', 'liver'*	*'Belly', 'tummy', 'stomach', 'throat', 'spleen'*
Symptoms	*'Sore', 'hurt', 'tired', 'sick', 'I don't want to eat'*	*'Achy', 'sore', 'hurt', 'sharp', 'burn', 'tight band squeezing me'*
Medical Terminology	*'Cannula', 'liver transplant', 'septic', 'scar', 'biopsy', 'IVs'*	*'Diuretics', 'portacath', 'cannula', 'potassium', 'haemorrhaging', 'broncoscopy', 'spasm', 'portal hypertension', 'upper right quadrant'*
Comparatives, superlatives and assessments of health status	*'Really poorly', 'not really poorly', 'sick', 'horrible', 'weak', 'a bit', 'a bit but not that much', 'a little', 'a lot', 'quite a lot', 'more', 'worse', 'better'*	*'Feeling well', 'not feeling well', 'rubbish', 'poorly', 'weaker', 'worn out quicker', 'not 100%', 'not really up to doing anything', 'not well enough', 'worse', 'worst', 'quite a lot', 'made me go down hill'*
The condition as a series of losses		
Description of losses	*'Couldn't even move', 'couldn't move my arm', 'the one thing I can't do anymore', 'trouble keeping up with my schoolwork', 'I'm not allowed to eat or drink certain things'*	*'I can't really do anything… well in terms of like other people your age', 'can't do as much as other people', 'I miss out', 'can't really have that much privacy', 'can't go to school by myself', 'get back to like me playing football'*
Being the sick one		
Comparison with others	*'I am the big brother… But he's taller'*	*'I don't want them to see me as the sick (child name) and the one that isn't able to do stuff'*
Markers of difference	*'Lost my hair', 'my tummy was big', 'my swallowing tube'*	*'Finger pricker thing', 'horrible medicines', 'injections', 'have operations', 'lost the lower part of my leg'*
Emotional impact	*'Like I could choke on baked beans because of my swallowing tube. And my feelings are happy when people are nice and caring and sad when people are mean and tease me'*	*'I mean confidence wise it makes me feel bigger because I'm…I'm more enlarged around the middle and it's just like my self-confidence goes… '*
