# Supplementary material for: ‘My life is a mess but I cope’: An analysis of the language children and young people use to describe their own life-limiting or life-threatening condition
Source: Palliat Med. 2024 Mar 4;38(3):379–88. doi: 10.1177/02692163241233977 (PMC10973786; doi:10.1177/02692163241233977)
Supplement: sj-pdf-2-pmj-10.1177_02692163241233977 – Supplemental material for ‘My life is a mess but I cope’: An analysis of the language children and young people use to describe their own life-limiting or life-threatening condition [file sj-pdf-2-pmj-10.1177_02692163241233977.pdf]

[Parent/carer to give additional information for questions below]

Introduction

Thanks for seeing me today. My name is XXX.

We can play, draw can talk together. XX (PARENT/CARER) will be with us as well.

I spend lots of time talking and playing with different children. While we are playing together I would really like to find out more about what you like doing, and what makes you feel happy.

If you get tired or want to take a rest please tell me. Is that OK?

We can start now and I will switch on this machine. It will keep what we have talked about so that I can listen to it later so that I can think about what you have said.

Guide

First of all I'd like to get to know you a little better. Can you tell me how old you are? And what do you enjoy doing? What kinds are things do you think are fun? Is there anything you'd like to know about me?

That's great- now I'd like you to tell me about when you see the doctors and nurses. What sort of things you tell them about?

Do you have anywhere in your body that hurts you? Where on your body, how does if feel?

And how does that make you feel?

How about inside, do you ever feel worried about anything? Do you tell anyone?

Do you like going out to play? Who with? Where do you like to go? Does feeling poorly ever stop you from playing?

Of all the things you've just told me, what do you think matters most to you? What would you like doctors and nurses to do for you to help you?

Does anybody ever ask you about that? Would you like them to? Would it be OK if a doctor or nurse asked you about these things?

Who would you like to ask you (prompt staff, family others)?

If someone was feeling poorly how would you tell them? What do you say to tell a grown up so that you know that you are feeling poorly?
