# Supplementary material for: ‘My life is a mess but I cope’: An analysis of the language children and young people use to describe their own life-limiting or life-threatening condition
Source: Palliat Med. 2024 Mar 4;38(3):379–88. doi: 10.1177/02692163241233977 (PMC10973786; doi:10.1177/02692163241233977)
Supplement: sj-pdf-4-pmj-10.1177_02692163241233977 – Supplemental material for ‘My life is a mess but I cope’: An analysis of the language children and young people use to describe their own life-limiting or life-threatening condition [file sj-pdf-4-pmj-10.1177_02692163241233977.pdf]

KING'S College LONDON

Introduction

Thanks for seeing me today. We can talk and we can also play together if that would help, and I'll ask you to tell me about the things that matter to you and what are the things you care about when you are feeling poorly. We can talk, play and draw to help you tell me if that would be easier for you. Your (PARENT/CARER) can join us as well if you want. If you get tired or want to take a rest please tell me. Is that OK?

We can start now and I will switch on this machine.

Guide

First of all I'd like to get to know you a little better. Can you tell me how old you are? And what do you enjoy doing? What kinds are things do you think are fun? Is there anything you'd like to know about me?

That's great- now I'd like to learn a little more about your illness. What sort of things does it do to you- probe:

Any problems it gives with your body? Where on your body, how does if feel?

And how does that make you feel?

How about inside, do you ever feel worried about anything? Do you tell anyone- why?

How about how your illness affects other parts of your life, like seeing friends, going out to do things, school? Can you tell me about your friendships- what is good about them and what could be better? What could help you with your social life?

What about the things you might wonder about- do you have questions about your illness and how you are cared for? Although I am not the right person to answer them for you, I'd like to know what your questions might be? Have you tried asking anyone? Why?

Of all the things you've just told me, what do you think matters most to you? Anything else?

Does anybody ever ask you about that? Would you like them to?

Do you think it would help if your care team (doctors, nurses and others that help look after you) asked you about some of the things you have told me about? Would it be OK if they asked you each time they saw you? Or how often? And if they asked you about a problem, how far back do you think it would be best to remember, so perhaps how has this bothered you in the last day, 3 days, week- what is easiest to remember?

Who would you like to ask you (prompt staff, family others)?

And who could have that information you share about the things that bother you and matter to you- just your care team, you, your family? Why?

I'd really like to hear your ideas about how we could best measure what matters to you, if we were trying to find out how much of a problem something was for you from maybe not at all to bothering you very much, how could we ask that? Some ideas might be pictures showing things getting better or worse, or numbers from very low to high, you could mark it with a pen, you could do it on an ipad screen- what do you think you'd like to do?

The last thing I'd like to ask is what you think would be a good way to ask someone in a sentence if they are feeling better or worse than the last time they saw their team- what could we ask?

Is there anything else you'd like to ask me or tell me?

Thank you.
